# General public’s understanding of rare diseases and their opinions on medical resource allocation in Japan: a cross-sectional study

**DOI:** 10.1186/s13023-023-02762-x

**Published:** 2023-06-08

**Authors:** Haruka Nakada, Saori Watanabe, Kyoko Takashima, Shohei Suzuki, Yuki Kawamura, Yutori Takai, Kenji Matsui, Keiichiro Yamamoto

**Affiliations:** 1 Division of Bioethics and Healthcare Law, Institute for Cancer Control, National Cancer Center, 5-1-1 Tsukiji, Chuo-Ku, Tokyo, 104-0045 Japan; 2 Department of Public Policy, The Institute of Medical Science, The University of Tokyo, Minato-ku, Tokyo Japan; 3 Bioethics Section, Center for Clinical Sciences, National Center for Global Health and Medicine, Shinjuku-ku, Tokyo Japan; 4 Graduate School of Social Sciences, Hitotsubashi University, Kunitachi, Tokyo Japan; 5 Faculty of Informatics, Gunma University, Maebashi, Gunma Japan

**Keywords:** Ethics, Public survey, Rare diseases, Resource allocation

## Abstract

**Background:**

Rare diseases (RDs) may impose a considerable financial burden on patients and their families. Public acceptance is essential to ensure sustainable public systems supporting RDs, especially in countries with universal healthcare coverage, such as Japan. This study aimed to explore the public’s understanding of RDs and identify crucial factors associated with the public acceptance of prioritizing financial support for RDs in Japan.

**Methods:**

An online questionnaire was sent to 131,220 Japanese residents aged 20–69 years. The items included in the questionnaire were general interest in medical science and medical care, general knowledge regarding RDs and health care systems, opinions on the cost of medical care, opinions on the research and development of RDs and common diseases, and individual characteristics.

**Results:**

The responses of 11,019 respondents were analyzed. Several respondents agreed to partially cover the medication cost of adult and pediatric RDs (59.5% and 66.8%, respectively) with public funding. The major reasons for agreeing were the huge financial burden imposed on patients and their families, limited available treatment options, effects of RDs on the life planning of patients, and difficulties caused by RDs in the patient’s social life. Furthermore, the respondents ranked RDs (56.0%) higher than common diseases (44.0%) for government funding for research and development. The reasons for supporting government-funded research and development for RDs included the lack of treatment options for numerous RDs (34.9%) and difficulty of studying RDs owing to the small number of researchers (25.9%). The chief reasons for supporting government-funded research and development for common diseases were the large number of affected patients (59.7%) and the possibility of more treatment options becoming available through the promotion of research and development (22.1%).

**Conclusions:**

The general public considers burdens associated with daily living or finance more than the epidemiological characteristics of RD while making funding decisions, demonstrating that rarity was less prioritized. A gap appears to exist between the general public and RD experts regarding the understanding of the epidemiological characteristics of RD and its thresholds. This gap should be bridged to ensure that prioritization of financial support for RDs is accepted by the society.

## Background

Rare diseases (RDs) affect a smaller patient population compared with common diseases. RDs can lead to several issues, including financial concerns due to the small market. The cost of treatment for each patient with RD increases with increasing research and development cost for RDs. The cumulative financial burden on patients with RDs and their family members has been a major problem; thus, several countries have public systems for providing financial support for RDs. For example, Japan has universal healthcare coverage (UHC) and additional public financial support based on the Act on Medical Care for Patients with Intractable Diseases. Concerning cost-effectiveness, the justification for prioritizing RD over common diseases is often controversial. Basing funding decisions on the high valuation of the health outcomes of a condition just because it is rare appears unsustainable and incompatible with other equity principles and theories of justice [1].

For the development and sustenance of public systems, the prioritization of RDs in financial support allocation requires public acceptance, especially in countries with UHC. In Canada, the public prioritized Improved Quality of Life and Effective Health Care for making decisions related to the reimbursement of money spent on RD drugs [2]. In a previous study, rarity was ranked the lowest among 13 items, which included Quality of Life, Severity, and Ability to work, for drug coverage decision-making [3]. In the UK, a proposal stating that the cost-effectiveness of funding decisions for RD healthcare should be treated differently from that for the healthcare of other diseases has been controversial. In Norway, general support has been provided for the equal rights of patients for treatment despite the higher cost of RD healthcare [4]. There was little evidence of a societal preference for rarity when considering treating patients with RDs at the expense of treating those with common diseases.

Japan, which has UHC, has also been struggling with issues related to healthcare coverage for high-priced drugs for RDs. For example, onasemnogene abeparvovec (Zolgensma), a spinal muscular atrophy gene therapy, which also happens to be the most expensive medicine covered by national health insurance, costs ~ 167 million yen ($1.56 million) per patient [5]. When a high-priced drug is introduced to the market, numerous issues, such as the price decision-making processes, cost-effectiveness assessment, and public understanding, emerge. In countries with UHC, medical costs are covered by citizens’ taxes and/or insurance premiums. Given that health care is recognized as a public good in these countries, an understanding of public opinion is important to justify the prioritization of RDs in allocating financial support.

This study aimed to reveal the public understanding of RDs and identify the crucial factors that lead to public acceptance of the prioritization of RDs in allocating financial support for healthcare. Furthermore, we sought to discuss the awareness of the general public of Japan regarding RD when they were asked about their views regarding the healthcare funding decision-making by the Japanese government in reference to other countries.

## Methods

### Survey design and participants

This cross-sectional survey was conducted via an online platform provided by INTAGE Inc., Japan, a marketing research company. INTAGE Inc. has a pool of ~ 10 million potential Japanese respondents for various surveys. We invited participants between the ages of 20 and 69 years from this pool. Quota sampling was employed in the recruitment process. The distribution of the sample by sex, age group, and residential area was similar to that of a representative Japanese population. This distribution was based on statistics from the national census. We sent an anonymous, quantitative, and self-administered online questionnaire to 131,220 registered Japanese residents. From January 26 to 31, 2022, we obtained responses from 11,019 individuals. INTAGE Inc. created web pages for recruiting volunteers from the pool of targeted residents, collected responses, and then sent us the dataset including each response while maintaining patient anonymity. The questionnaire was designed to elicit an online reply after each potential respondent had read an explanation of the study’s purpose. Only complete responses were considered in the data analysis.

### Questionnaire and analysis

The questionnaire was developed based on discussions among the authors. First, we reviewed relevant surveys regarding public attitude toward reimbursement decision-making for RD drugs or public views on orphan drugs as well as other papers discussing RD-related issues [4, 6–9]. Next, we extracted and developed the items according to our research objectives. After developing a draft, we conducted a pretest wherein five colleagues who were not in the research team participated to help make the questionnaire clearer and more comprehensive. The questionnaire contained a maximum of 29 closed questions and Likert scale questions. It included five sections: (A) general interests in medical science and medical care (three items), (B) general knowledge regarding RD and healthcare systems (three items), (C) opinions on expenses for receiving medical care (15 items), (D) opinions on research and development on RDs and common diseases (three items), and (E) individual characteristics (four items).

Section A aimed to identify the interest of the respondents in the topics of medical science and medical care, the sources of their information, and their experience of being affected by RDs. Section B asks regarding the RD features that the respondents understood and the number of patients with RDs estimated by them. This section evaluated the qualitative and quantitative perception of the respondents regarding RDs. In section C, the respondents were asked about their opinion regarding sharing the RD medical cost with the public. We included questions such as “How do you feel about your insurance premiums and taxes being used to cover a part of the cost of healthcare for these [adult or pediatric] rare diseases?” Furthermore, they were asked regarding the conditions required to cover the high medication cost for RDs by their insurance premiums and taxes as well as the ideal mechanism to cover the medication cost for RDs other than the UHC. Section D asked about the financial resource allocation among RDs or common diseases; the question was as follows: “If the government provides financial support for research and development, which area do you think should be given more attention: common diseases such as lifestyle-related diseases or rare diseases?” Section E asked regarding the profiles of respondents, such as occupation, educational background, and current health conditions.

Descriptive statistics and the chi-squared test were used for data analysis. The opinions of the respondents regarding covering the RD medication cost were compared between adult RD cases and pediatric RD cases. Two-sided* p*-values of ≤ 0.05 were considered statistically significant. All data were analyzed using SPSS (version 25.0).

## Results

### Respondents’ characteristics

We analyzed 11,019 respondents (Table 1), 1087 (9.9%) of whom had experiences with RDs, as they or people close to them had been affected by RDs (defined here as “RD-experienced respondents”). Television (75.1%) was the major source of information regarding health care or medical science followed by the Internet, except for social networking services (SNS) (60.9%). Most respondents (77.7%) had interests in healthcare or medical science topics to varying degrees.

**Table 1:** Respondents’ characteristics

N = 11,019	n	%
*Age*		
20–29	1915	17.4
30–39	2009	18.2
40–49	2568	23.3
50–59	2430	22.1
60–69	2097	19.0
*Average age*		
45.3		
*Sex*		
Male	5746	52.1
Female	5236	47.5
Others	37	0.3
*Occupation*		
Full-time employee	4905	44.5
Part-time employee	1977	17.9
Independent business owner	714	6.5
Student, housekeeper, unemployment	3423	31.1
*Education*		
Junior high school	212	1.9
Senior high school	3438	31.2
Technical school	1071	9.7
College	1352	12.3
University	4085	37.1
Graduate school	458	4.2
N/A	403	3.7
*Marital status*		
Married	5804	52.7
Not married	5215	47.3
*Children whom I live with*		
No	7628	69.2
Yes	3391	30.8
*Household income*		
< $200,000,000	1066	9.7
$200,000,000–$299,999,999	1003	9.1
$300,000,000–$399,999,999	1097	10.0
$400,000,000–$499,999,999	1090	9.9
$500,000,000–$599,999,999	984	8.9
$600,000,000–$699,999,999	639	5.8
$700,000,000–$799,999,999	604	5.5
$800,000,000–$899,999,999	386	3.5
$900,000,000–$999,999,999	416	3.8
≥ $1,000,000,000	774	7.0
I do not know/I do not want to answer	2960	26.9
*Current health condition*		
Going to the hospital now	3979	36.1
Went to the hospital during the past 3 months	1011	9.2
Went to the hospital during the past 6 months	488	4.4
Went to the hospital last year	650	5.9
Did not go to the hospital last year	4891	44.4
*I and/or the people around me experienced a rare disease*		
Yes	1087	9.9
No	9075	82.4
I do not want to answer	857	7.8
*Source of information on healthcare and medical science*		
Television	8271	75.1
Internet other than SNS	6713	60.9
Newspapers	2997	27.2
Word-of-mouth communication	2588	23.5
SNS (e.g., Facebook, Twitter, Instagram, TikTok)	2059	18.7
Magazines	1422	12.9
Radio	1159	10.5
Newsletter, free news magazine	527	4.8
Scientific articles	325	2.9
N/A	999	9.1
*Interests in topics of healthcare and medical science*		
I am interested in the topics	1853	16.8
I tend to be interested in the topics	6712	60.9
I tend not to be interested in the topics	1992	18.1
I am not interested in the topics	462	4.2
*Assessment of the progress of healthcare and medical science*		
There are more positives	2127	19.3
Somewhere more positives	4784	43.4
Both are the same	3606	32.7
Somewhere more negatives	341	3.1
There are more negatives	161	1.5

*SNS* social networking services

### Understanding of RD: qualitative and quantitative perceptions

**Fig. 1:**
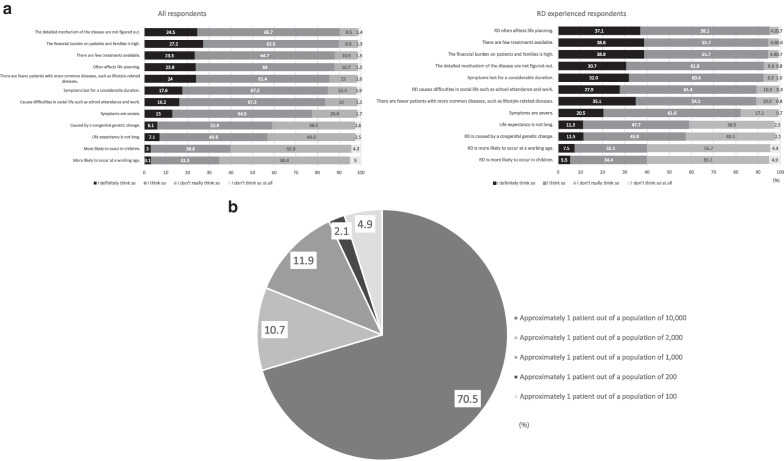
**a** Qualitative evaluation of rare diseases. The illustration shows certain features of rare diseases (RDs) ranked by the percentage of RD-experienced respondents who answered “I definitely think so” and “I think so” about the feature in question. Abbreviation: RD, rare disease. **b** Quantitative evaluation of rare disease. The estimation of the respondents of the number of patients with rare diseases

The respondents were asked regarding their qualitative and quantitative perceptions of RDs (Fig. 1a, b). Figure 1a shows the ranking of RD features by the percentage of “I definitely think so” and “I think so.” Uncertainty regarding the detailed disease pathogenesis, financial burden on patients and their families, and paucity of available treatments were the most commonly known features of RD. Conversely, features such as the cause of RDs (“RDs are caused by congenital genetic changes”), shortened life expectancy, and age of onset (“RDs are more likely to occur in children” and “RDs are more likely to occur at a working age”) were the least recognized. The respondents thought the number of patients with RDs was beneath the threshold for the definition of “rare diseases” in the EU, Japan, and the US (Fig. 1b). In the EU, orphan medicinal products are intended for the diagnosis, prevention, or treatment of life-threatening or very serious conditions that affect no more than 5 in 10,000 people [10]. In Japan, patients with specific diseases whose prevalence is only ~ 0.1% can receive financial support according to the Act on Medical Care for Patients with Intractable Diseases. Furthermore, the Act on Securing the Quality, Efficacy, and Safety of Products Including Pharmaceuticals and Medical Devices defines an orphan drug as a drug for a disease affecting < 50,000 patients. In the US, the Orphan Drug Act defines an RD as a disease or condition that affects < 200,000 people [11]. Our survey revealed that ~ 70% respondents declared that patients with RDs account for only 0.01% of the population, which is smaller than the RD definition in the abovementioned orphan drug policies (Fig. 1b), whereas > 80% respondents thought that RD cases were fewer than the cases of common diseases (Fig. 1a).

The RD-experienced respondents had a higher percentage of “I definitely think so” in each feature than all the other respondents. Moreover, they had a poorer understanding of the features relevant to reduced life expectancy, causes of RDs, and age of onset of RDs; this tendency was similar to that of the entire respondent group.

### Attitudes toward public funding of a part of the cost of RD medication

**Fig. 2:**
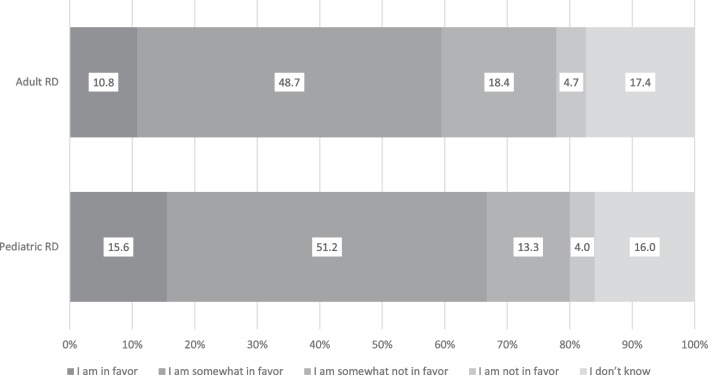
Opinions of the respondents regarding the coverage of medication costs by insurance premiums and taxes. The respondents’ opinions regarding whether they were in favor of insurance premiums and taxes covering medication costs for adult and pediatric rare diseases. Abbreviation: RD, rare disease

Most respondents (75.5%) considered that the current medical expenses were a heavy burden in general. Figure 2 shows the respondents’ opinions regarding covering a part of the medication cost for RDs using public funds, including insurance premiums and taxes paid by them. Pediatric RDs received significantly higher favor (66.8%) than adult RDs (59.5%) (Fig. 2, *p* < 0.01). Among the RD-experienced respondents, “I am in favor” and “I am somewhat in favor” for adult RDs accounted for 19.9% and 56.0%, respectively, whereas those in pediatric RDs were 25.9% and 55.4%, respectively. The percentage of “I do not know” responses was slightly higher for adult RDs than for pediatric RDs (8.5% vs. 6.8%).

The major reasons why the respondents agreed with the partial coverage of the RD medication cost by public funding were as follows: huge financial burdens on patients with RDs and their families, limited available treatment options, the fact that RDs often affect the patient’s life planning, and because RDs cause difficulties in social life, including learning or working opportunities. These reasons tended to be similar for adult and pediatric RDs. Conversely, only a few respondents selected the following reasons: shortened life expectancy, RDs are caused by congenital genetic changes, and population of patients with RDs is less than that of patients with common diseases (including lifestyle-related diseases). Regarding the opinions of respondents about adult RD, few respondents selected the reason “RDs are more likely to occur at a working age.”

**Fig. 3:**
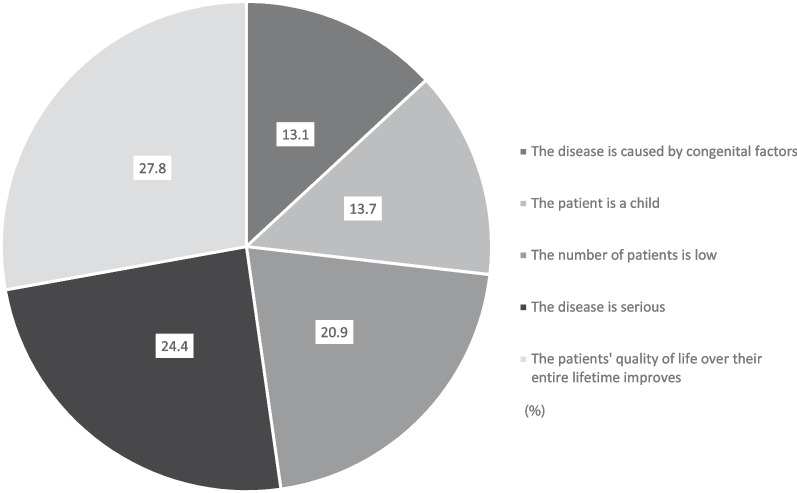
Conditions required for the partial coverage of high-price rare disease medications. The respondents’ opinions regarding which conditions should be considered for the partial-cost coverage of high-price medications, such as Zolgensma

Furthermore, the respondents selected the most necessary conditions that should be considered if high-priced RD medication were to be partially covered by public funding (Fig. 3). Zolgensma was held considered as an example of an RD medication with high cost.

### Mechanism of sharing the RD medication cost

**Fig. 4:**
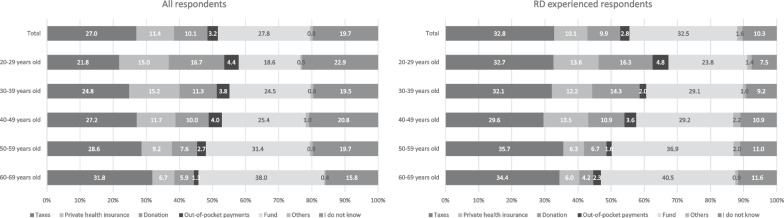
Respondents’ opinions regarding cost coverage for rare disease medications in addition to universal healthcare coverage. The respondents’ opinions regarding which organization should cover the cost of medications for rare diseases in addition to universal healthcare coverage. All he respondents and those who experienced rare diseases are shown for comparison. Abbreviation: RD, rare disease

The respondents selected the most ideal mechanism (other than UHC) to cover the RD medication cost (Fig. 4). Many of them selected “fund” (27.8%) or “taxes” (27.0%). “Fund” is defined as “jointly funded by pharmaceutical companies and the government,” and “taxes” as “paid by eligible people (e.g., consumption, income, and residential taxes).” More respondents of younger ages selected individual payment, including private health insurance and donation, than those of older ages.

Among the RD-experienced respondents (Fig. 4), the rate of “I do not know” responses was 10.3%, which was nearly half of that in the entire respondent population. Many of the RD-experienced respondents selected “taxes” (32.8%) or “fund” (32.5%), similar to the other respondents.

### Financial resource allocation for medical research and development

**Fig. 5:**
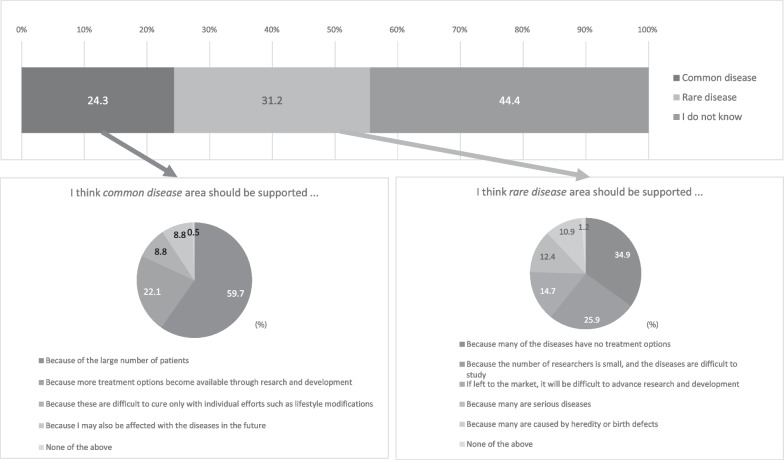
Disease areas that the respondents considered should receive the government’s financial support and reasons for the same. The respondents’ opinions regarding which areas of disease research and development should be financially supported by the government. Each respondent selected only one reason for his/her opinion

More than 40% respondents responded, “I do not know” whether the government financially supports research and development of common diseases or RDs. (Fig. 5). When omitting this response, the proportion of respondents who selected RDs and common diseases was 56% and 44%, respectively. Among the RD-experienced respondents, the proportion of those who supported RDs was higher than that of those who supported common diseases (60.6% vs. 39.4%, without the “I do not know” response) for financial resource allocation for medical research and development.

The reasons for the provision of financial support for RDs by the government were as follows: numerous RDs have no available treatment options (34.9%) and little is known regarding RDs owing to the small number of studies conducted on them (25.9%). As for common diseases, the main reasons were as follows: large patient populations (59.7%) and more treatment options becoming available through research and development (22.1%).

## Discussion

This survey revealed the qualitative and quantitative perceptions of Japanese citizens regarding “rare diseases.” The study discussed the factors these citizens focused on when justifying the prioritization of RDs in allocating financial support. Although most respondents were not aware of the epidemiological features of RDs, they clearly understood the RD-related difficulties faced by patients and their family members and RD research and development. Regarding the quantitative perceptions of RDs, although most respondents recognized that the number of patients with RDs was lower than that of patients with common diseases, the quantitative estimation of the respondents of the number of individuals experiencing RDs differed from the number defined in the country's orphan drug policies.

### Understanding regarding RDs

This survey revealed that the epidemiological features of RDs, such as genetic mutations as their pathogenesis, age of onset, and shortened life expectancy, were not known by most people. The Japanese government or agencies often employ the term “intractable disease” [12], which includes “rare disease” in their policy papers. Therefore, Japanese citizens may tend to regard RDs as conditions that have something to do with intractability. The respondents recognized the difficulties in RD research and development and those closely associated with the lives of patients with RD. As most people experience being sick and having some difficulties in daily life at varying levels, they may be more familiar with recognizing treatment- or daily life–related RD issues than those associated with the epidemiological features of RDs. Conversely, researchers and physicians usually focus on the epidemiological characteristics of RDs when conducting research and treating patients with RDs [12]. Hence, a gap appears to exist regarding the focus on the epidemiological characteristics of RDs between the general public and RD experts. This gap may lead to an inadequate understanding of RD research and development and a low acceptance of financial support for RD treatment and research.

When describing “rare diseases,” people tend to think of “rarity,” which signifies that patients with RDs are considerably fewer than those with common diseases. Most respondents thought that the number of patients with RD is smaller than that described in the definition of RDs in orphan drug policies in the EU, Japan, and the US. People appeared to be unfamiliar with RDs owing to the small number of patients experiencing them. This observation could be one of the reasons why the epidemiological characteristics of RD are difficult for them to understand. According to a previous study that examined 32 countries and regions, the average prevalence of RDs defined by each organization was 40–50 cases per 100,000 people. It is widely acknowledged that there are various quantitative definitions of RDs that are recognized internationally [13]. Given the lack of a unified quantitative definition of RDs even among experts, it is understandable that the general public may not have a precise understanding of the quantitative definition of RDs. Rather than scientific discussions such as those regarding disease prevalence, the word “rare” for RDs in Japan has been defined based on political discussions, such as government financial assistance for patients and promotion of research and development. Thus, a gap also appears to exist between the definition of RDs in policies and the public perception of a “rare disease.”

### Public attitudes toward the resource allocation of funds for medication and research and development in RDs

In this survey, 75.5% respondents felt they bore a heavy burden of medical fees in general, which is similar to the findings of a previous study conducted in Japan [14]. However, the general public may understand the financial issues that patients with RD and their family members face according to their personal experience. Having RD-related experience was one of the crucial factors in forming one’s opinion regarding sharing the cost of RD medication and research and development. Through their RD-related experiences, these individuals gradually understood the importance of financial support for RD medicine or research and development and accepted the prioritization of RDs.

Our respondents appeared to accept the prioritization of RDs in the allocation of financial support in light of their understanding of RDs. However, they did not consider the RD feature “RD is caused by a congenital genetic change” and the age of onset, which were low in the ranking of public perception for supporting the prioritization of financial support for RDs. Furthermore, between the adult and pediatric RDs, people preferred sharing the medication cost of pediatric RDs through public funding. The age of onset or patients’ age could explain the difference in attitude among the responders. Younger patients generally attract more attention or sympathy than older patients, leading to a higher acceptance of the prioritization of financial support for pediatric RDs. Recent news articles have reported that high-priced RD medications, such as nusinersen (Spinraza) and Zolgensma, have been administered to pediatric patients. The fact that the media propagates such information could further attract the attention of the public toward pediatric RD medication costs.

For sustainable financial support for patients with RDs, we need to discuss alternative mechanisms to UHC. Although 24.7% respondents selected individual payment, several individuals expected public funding, including taxes or government funding, to support these patients. Pharmaceutical companies were also expected to play a role in financially supporting patients with RDs. For instance, these companies accept “compassionate use” requests for orphan drugs. This form of assistance tends to fit squarely within corporate social responsibility programs. When developing cofunding by the government and pharmaceutical companies to financially support patients with RDs, two challenges include agreement within companies regarding providing the fund according to their business decision and the expectation of a return on investment. The findings of this survey revealed that younger respondents tended to prefer individual payment (private medical insurance, donations, or out-of-pocket costs) as an alternative to UHC for RD medication. As people age, they generally have more disease-related experiences and often use healthcare services. According to these experiences, older individuals may recognize health care as a public good or a public service, especially in Japan and other countries with UHC. Conversely, people who prefer individual payment may recognize health care as a commodity that the customers pay for according to their needs [15]. If the younger generation’s preference (individual payment) continues in the future, the policies or social systems promoting the commodification of healthcare may become publicly supported; however, they may cause inequity in healthcare access among patients with RDs. Another reason for the preference of individual payment for RD care can be that RDs are often associated with personalized medicine [16, 17]. Although the exact cause for many RDs remains unknown, it can be traced to mutations in a single gene. RD research is now fulfilling the promise of targeted therapy that is sometimes applied to a single patient. For example, in 2019, the US Food and Drug Administration permitted “N = 1” or “individualized” therapy, known as “milasen,” for Batten’s disease [18, 19]. Considering that personalized or individualized therapy is often applied to only a few patients, it appears more suitable for individual payment rather than public funding.

### Factors favoring priority funding for RD: rarity or otherwise?

A typical feature of RDs is that fewer patients experience them compared with common diseases. Thus, survey demonstrated that disease rarity slightly influenced the respondents’ opinions regarding whether they agreed to cover the cost of RD medication partially with public funding. In line with this finding, several previous studies argued that among several factors, rarity should not be highly valued when allocating healthcare resources. Regarding public opinions toward decision-making on insurance coverage for RD drugs, the public previously ranked “rarity” the lowest among the factors to be considered [3, 8]. The opinions of the general populace ought to be considered when formulating policies regarding the allocation of public funds toward the treatment of RDs and common diseases. However, we found insufficient evidence that societal preference for rarity exists in the treatment of patients with RDs at the expense of the treatment of those with common diseases [4]. In a policy debate in the UK, there were controversies regarding the justification of the special status of RDs and whether the cost-effectiveness of drugs for RDs or ultra-RDs should be considered differently from that of other drugs and interventions. Valuing health outcomes more highly because of the rarity of the condition appears unsustainable and incompatible with other equity principles and theories of justice [20]. Magalhaes argued that the severity of a disease should be considered more than its rarity when setting priorities in orphan drug policies [21].

The findings of this survey suggested several factors other than rarity for the public acceptance of the prioritization of RDs in the allocation of financial support. Previous studies have also reported that the determinants of social preference for orphan drugs are complex. For example, a survey of the French public identified nine factors that determined the value of orphan drugs, including disease-associated disability [22]. The factors in this survey can be categorized as “RDs’ epidemiological characteristics” and “burdens on daily living.” The most highly prioritized factors were “burdens on daily living,” including financial burdens on patients and their family members and affected life planning, whereas the least was “RDs’ epidemiological characteristics,” such as shortened life expectancy and the genetic etiologies of RDs. Previous research regarding the treatment of short life expectancy appears to differ from the results of our study. The study conducted in the UK reported a strong preference for treatments that improved the quality of life of patients and prolong their survival after indicating that the general public did not consider the rarity of the disease in itself sufficient to justify special preferential treatment in the NHS [23]. The French study also suggested that the rationale for public funding of orphan drugs is that people value the impact of drugs on life expectancy, especially the significant extension of life expectancy, more than their impact on the quality of life [22]. In other words, prolonging the life expectancy of patients is an important factor in justifying preferential financial support for RDs in these studies. Although the methods used in their survey differ from those used in the present study, it can be argued that in Japan, the burden closely related to the very-short-term impact on life was a more important factor considered by the citizens than the long-term impact on people’s whole life.

The financial support for pediatric RDs, which starts at a very early age, is more acceptable for the public, probably because the public feels more sympathy for younger patients or attaches a considerable value to the potential life expectancy. Furthermore, our respondents prioritized the improvement of the quality of life of patients or mitigation of disease severity higher than the rarity of the disease when considering partial coverage of high-price RD medications.

### Funding for RD research and development

In this survey, no less than 40% of the respondents responded “I do not know” for whether the government should support funding research and development for an RD or a common disease. The fact that a certain percentage of people did not select between RDs and common diseases is consistent with the findings reported by previous studies as follows. In Canada, a survey that asked citizens aged ≥ 19 years to select between subsidizing the treatment of patients with rare or common diseases under different scenarios reported that 23.8–30.4% respondents expressed indifference [24]. In Norway, similar surveys involving 40–67 year-old participants revealed that 65% [4] and 75% [25] expressed indifference. Their attitudes may be explained by the following three points. First, they did not have adequate experience with or information regarding drug development. Given that research and development could be more unfamiliar to the general public than medical care, they might not have had any idea regarding what should be considered for funding allocation. This argument can be supported by the findings of a previous study that reported that the lack of knowledge and involvement in the issue of orphan drugs and common disease drugs may result in the lack of prioritization of both [24]. Second, RDs have been given less attention than common diseases by the general public. Although the total number of patients with RDs is increasing in Japan and elsewhere, the number of individual RD cases remains small. Therefore, it could be more difficult for the respondents to connect the funding issue with the general public’s individual problem. Third, the respondents cannot prioritize the two because they consider both important. Regardless of whether the disease is rare or common, spending a lifetime as a patient lead to various levels of burden. The respondents who responded “I do not know” may make disease types irrelevant to funding. Public preferences for orphan drug funding are often examined in a “zero-sum” framework. It has been suggested that this framework may miss important elements of public preferences for orphan drug funding [24]. Even in this survey, only the intentions of those who indicated their willingness to vote are represented in the survey results, and the different societal preferences would be expressed if the intentions of the ~ 40% who avoided voting were expressed.

Over 30% respondents supported the prioritization of RDs in the allocation of financial support for research and development, suggesting that such support is accepted at least to a certain degree. Moreover, excluding those with the “do not know” response, there were more respondents (56.2%) who supported government funding for RDs than those who supported government funding for common diseases. In previous studies, the public favored treating RDs over treating common diseases or prioritized not increasing the size of the waiting list [23]. The main reasons for the acceptance are related to RD features such as the availability of only a few treatment options or the existence of difficulties in promoting RD research, which were perceived frequently by the respondents. Other reasons were related to the epidemiological characteristics of RDs, severity of the disease condition, and hereditary cause of the disease. The general public tended to make funding decisions in medical research and development based on their understanding of RDs.

### Implications and future perspectives

This survey has several implications and future perspectives. First, healthcare policies or systems should focus on the unapparent burdens of RDs. When considering policies to support RD care, focusing solely on rarity may lead to a limited focus on the quantifiable burdens of patients and their family members. Numerous respondents of this study were broadly aware of the factors related to the quality of life of patients with RD. It may be desirable to discuss RD policy with all stakeholders, including patients and citizens, in light of the qualitative perceptions of the citizens and the concept of “intractability” [12], which has often been referred to in Japanese policy. Given that the burdens on patients with RDs and their family members are often uncountable and vague, the policies or systems should cover these burdens appropriately. The general public’s decision regarding funding for RD healthcare and research and development appears to be supported by ethical principles, such as the “sickest first” or the “rule of rescue,” rather than rarity. According to the “sickest first” principle, medical resources should be allocated to the sickest people regardless of their prognoses [26]; according to “the rule of rescue,” individuals identified to be in immediate peril should be rescued regardless of the cost [27]. These principles are categorized into so-called “prioritarianism,” wherein we should give greater weight to benefiting the worse off [28]. From another perspective, the general public may consider prioritizing a person in greater need for their funding decision.

Second, we need more social communication regarding RD-related information. Our survey revealed the gaps in the understanding of RD epidemiologic characteristics and threshold between the general public and RD experts. Such gaps in understanding may hinder the public from accepting the prioritization of RDs in financial support allocation. All stakeholders such as national agencies, medical/research institutions, pharmaceutical companies, and patient groups should acquire and disseminate sufficient information regarding RDs. Recently, some remarkable successes revealed that the promotion of RD research can also contribute to the development of drugs for common diseases. For example, the *PCSK9* gene effectively eliminates a protein in the blood that plays a fundamental role in controlling low-density lipoprotein levels. While this mutation causes a rare form of familial hypobetalipoproteinemia, the discovery of *PCSK9* led to the successful development of a drug that may help prevent cardiovascular diseases [29]. These facts would advance public understanding and their support for RD research and development. However, owing to the diversity of RDs, relevant information regarding these conditions would vary widely. Hence, developing an information toolkit for sharing common topics regarding RDs would be useful.

### Limitation

The main limitation of our study is that 70% respondents assumed a smaller patient population for the RD threshold in Japan and the EU and US. Thus, they may adopt different attitudes toward the prioritization of funding support for RDs when they understand the actual RD threshold.

## Conclusions

In making funding decisions for RDs, the general public considers burdens on daily living or financial burdens more than the epidemiological characteristics of RDs. Rarity, a typical characteristic of RDs, was less prioritized by the general public as a factor in making funding decisions. Conversely, a crucial factor for accepting the prioritization of RDs in the allocation of financial support might be the experience of experiencing an RD. However, a gap exists in the understanding of the epidemiological features of RDs and RD thresholds between the general public and RD experts. This gap should be bridged to achieve societal acceptance of the prioritization of RDs in the allocation of financial support.

## Data Availability

The datasets used during the current study are available from the corresponding author upon reasonable request. All data were developed in Japanese.

## Abbreviations

RD: Rare disease
UHC: Universal healthcare coverage
